# A critical analysis of research methods to study clinical molecular biomarkers in Endodontic research

**DOI:** 10.1111/iej.13647

**Published:** 2021-10-31

**Authors:** Matthias Zehnder, Georgios N. Belibasakis

**Affiliations:** ^1^ Clinic of Conservative and Preventive Dentistry University of Zürich Center of Dental Medicine Zürich Switzerland; ^2^ Division of Oral Diseases Department of Dental Medicine Karolinska Institutet Huddinge Sweden

**Keywords:** apical periodontitis, biomarker, dental pulp, methodology, root canal

## Abstract

The authors of this narrative review aimed to address various experimental methods and make recommendations for how research should move forward in the context of studying biomarkers in clinical Endodontic research. The approach adopted is exemplified using two prominent clinical problems, namely (a) the ‘reversible’ versus ‘irreversible’ pulpitis conundrum and (b) persistent idiopathic dentoalveolar pain (PIDAP). Pulpitis under deep caries or dentinal cracks is understood from a histological perspective, but clinical assessment tools to indicate irreversibly inflamed aspects of the dental pulp are elusive. PIDAP, on the other hand, is a diagnosis of exclusion; its pathophysiology is complex and not understood sufficiently to avoid unnecessary dental treatments. This review addresses how diagnostic biomarkers could further our understanding of those and other clinical problems, and how issues can be tackled from a methodological point of view. Hence, different methodological approaches to identify suitable diagnostic biomarker(s) or use known biomarkers are presented. The importance of asking a relevant research question, collecting the most suitable fluid and using the ideal collection vehicle for the research question under investigation is discussed based on the defined clinical problems.

## BIOMARKER RESEARCH IN ENDODONTOLOGY

The term biomarker has been defined as a characteristic that is objectively measured and evaluated as an indicator of normal biological and pathogenic processes, or pharmacologic responses to a therapeutic intervention (Atkinson & Colburn, 2001). The term is mainly used to describe a molecule collected from a biological fluid that is correlated to a specific condition, development of a condition or identification of the same. Hence, any type of study that identifies and correlates molecules from endodontic samples, such as biofilm components in symptomatic versus non‐symptomatic cases (Loureiro et al., 2021) could be seen as biomarker research. Moreover, the term ‘biomarker’ is also used in the context of imaging, and describes a biological feature or set of features in a diagnostic image, that can then be analysed using specific algorithms (Smith et al., 2003). This is also beyond the scope of this text, but represents an interesting and timely topic in Dentistry and Oral Medicine (Hung et al., 2020). For the sake of simplicity and focus, this text will follow the main topic of diagnostics, and how body fluid‐derived biomarkers can be used in that context in endodontic research. These methods focus on measurable host elements (e.g. biomarkers) that can be invariably linked to a disease or the outcome of a treatment (Naylor, 2003). It is also possible to identify a pattern of biomarkers that can be linked to a disease, a so‐called biosignature (Chen et al., 2020; MacLean et al., 2019). A useful way to classify biomarkers is thus to address their purpose (Winter et al., 2013) and the collection site (Fitzsimmons et al., 2010). This is summarized in Table 1. Other classifications of biomarkers exist (Naylor, 2003), but are not used in the current narrative review.

**TABLE 1:** Types of biomarkers that have been described and could be used in Endodontic research

Information	Systemic^a^	Local^b^
Diagnostic	Detect disease, its recurrence, or progression	
Prognostic	Provide insight into the natural history of disease (recurrence, survival)	
Predictive	Predict response to treatment	

^a^ Collected from peripheral blood.

^b^ Detected from a body fluid at or close to the site of interest, can be collected non‐invasively or intraoperatively.

It has long been suggested to use biomarkers to improve endodontic decision‐making (Mejare et al., 2012; Rechenberg & Zehnder, 2014), yet clinically useful tests remain elusive. The obvious research question relates to the choice of the best markers to identify a specific condition (Rechenberg et al., 2016). However, and beyond that, multiple other questions remain unanswered. The current text will use two clinical conditions to exemplify individual research topics that could further our understanding.

## WHY FOCUS ON CLINICAL STUDIES?

In theory, biomarker profiles could be studied *ex vivo*, for example in pulp‐dentine models or so‐called tooth slice models (Gonçalves et al., 2007; Magloire et al., 2001; Murray et al., 2008). These have their clear merits, as they can be used to assess reactions of resident cells to for example microbial challenges. However, such *ex vivo* models fail to simulate the clinical situations in the current context, because they lack innervation and blood supply. Therefore, they can for example neither simulate chemotactically attracted cells in pulpitis, nor nerve changes in persistent idiopathic pain, which are the two clinical examples used in the following text (see below). Because of the clearly clinical nature of diagnostic biomarker research in Endodontics, it would appear that the respective studies should be clinical in nature and performed in humans. Nevertheless, *in vitro* cell lines obtained from inflamed samples (Jin et al., 2018; Yonehiro et al., 2012) could be used to identify molecular markers (gene and protein) by candidate or profiling methods. Furthermore, rodent pulpits or reparative models could also be used as they possess a blood and nerve supply. Although we may wish to limit animal experimentation, they are commonly used for this purpose in science and do present an excellent pre‐human model.

## TWO POORLY UNDERSTOOD CONDITIONS

A simple way to identify conditions that are not properly understood or diagnosed in the field of Endodontology can be to look at ongoing discussions on disease terminology (Pigg et al., 2021; Rechenberg & Zehnder, 2020). In the context of pulpal and periapical disease, there are at least two issues that need further scrutiny: first is the question on ‘reversible’ versus ‘irreversible’ pulpitis in the context of minimally invasive endodontics (Careddu & Duncan, 2022; Wolters et al., 2017). Secondly, but not less clinically important, is persistent or ‘atypical’ tooth pain, recently termed persistent idiopathic dentoalveolar pain or PIDAP (Benoliel et al., 2020). Both conditions can be associated with severe pain, yet they are completely different in their nature. Pain is used by dentists as a core diagnostic criterion, as the patient suffering from a toothache expects pain relief. However, the crude approach of simply ‘drilling where it hurts’ will lead to overtreatment in the case of pulpitis (Careddu & Duncan, 2022), and false treatment in the case of PIDAP.

### Pulpitis

Pulpal and periapical inflammations that should be treated by the dentist/endodontist are related to non‐commensal biofilms growing in normally sterile spaces (Nair & Luder, 1985; Zehnder & Belibasakis, 2015). Because the pulp space and the periapical tissues form an anatomically connected unit, pulpal inflammation causes physiological alterations in the periapical tissues before the infection reaches the apical portions of the root canal system (Rechenberg et al., 2014). If not treated correctly, these conditions can lead to severe pain and systemic consequences such as hospitalization (Nalliah et al., 2011). Clinical assessment methods such as sensibility and percussion tests can give non‐conclusive results, especially in teeth with deep caries (Marending et al., 2016; Mejare et al., 2012).

### Persistent idiopathic dentoalveolar pain

On the other end of the clinical spectrum, ‘atypical’ tooth pain that is wrongly associated with infection/ inflammation of the pulp and periapical tissues can lead to false treatment decisions (Pigg et al., 2021), with the consequent untoward consequences for both dentist and patient (Feinmann et al., 1993). These conditions should specifically *not* be treated by the dentist, at least not by any form of operative intervention, but rather be referred to a pain specialist (Pigg et al., 2021). The question in the context of biomarker research is related to the targeted object of interest. Apparently, PIDAP affects a select group of patients (Policarpou et al., 2005). So, searching for systemic elements could be an approach for clinical research. Alternatively, alterations in the tissues surrounding the tooth could be investigated.

## WHY BIOMARKER RESEARCH COULD HELP

Diagnosing pulpitis and PIDAP remains challenging, despite considerable advances in imaging procedures over the recent years (ESE, 2019). As is discussed below, molecular diagnostics could indeed be used to further our understanding and possibly develop clinically useful chairside tests, and also gain insight into the origins of PIDAP.

Table 2 delivers an overview of these two clinical situations with focus on different approaches in endodontic biomarker research. The reason for the need of such extra information is the unique micro‐environment of the pulp space and the surrounding tissues. The dental pulp is a soft tissue encased in the hard tissue shell of the tooth. Because dental imaging methods are still based on radiography, they can merely detect changes in heavy elements, that is the relative calcium content (White & Pharoah, 2013), and are thus limited to mineralized tissue diagnostics. For this reason and with the current lack of soft‐tissue imaging in dentistry (Idiyatullin et al., 2011), the extent of soft tissue breakdown or pulpal biofilm infiltration cannot be assessed. Even clinically apparent inflammation in periapical tissues is not always readily detected on periapical radiographs, as it can occur at such a fast rate that its radiological depiction fails to reveal hard tissue alterations (Rechenberg et al., 2021). The direct intra‐dental assessment of bacterial penetration via fluorescent dyes and optical filters (Lennon et al., 2007) are also restricted to the dental hard tissue, and not practical in blood‐perfused organs such as the dental pulp.

**TABLE 2:** Two clinical conditions, pulpitis and persistent idiopathic dentoalveolar pain (PIDAP) and related research‐oriented questions/topics regarding biomarkers

Research topic	Pulpitis	PIDAP
Unknown issue	Level of microbial infiltration	Pathology
Biomarker type	Diagnostic/predictive	Diagnostic
Suitable fluid	Dentinal or pulpal? Pulpal blood or fluid? Plasma or serum?	Crevicular or periapical? Peripheral blood or saliva? Plasma or serum?
Local collection	Glass pipette or paper point?	Paper strip or paper point?
Target molecule	Single or multiple?	Single or multiple?
Internal control	Volumetric or total protein?	Volumetric or total protein?
Biosensor	Chair‐side or lab‐side?	Chair‐side or lab‐side?
Possible benefits	Identify irreversibly inflamed pulp tissue	Exclude periapical inflammation Identify pathological pathway

In the context of PIDAP, one clinically useful approach could also be to demonstrate the absence of inflammatory biomarkers in or around the tooth. It is a common clinical situation encountered by Endodontists that they receive a referral for a tooth, in which an endodontic treatment has been initiated, yet it does not stop hurting. In such a situation, assessing the inflammatory state of the periapical tissue could be extremely useful, also for the communication with the patient (Table 2).

## SYSTEMIC BIOMARKERS

Biomarkers detected by peripheral blood analyses can be utilized for monitoring general health (Table 1). Whilst this concept does not directly fall in the main topic of this review, it is worth being discussed briefly. Three types of systemic biomarkers have been named: diagnostic, prognostic and predictive markers (Winter et al., 2013). Systemic diagnostic biomarkers are mostly based on detection or quantification of protein or gene expression. Prominent applicable examples are cardiac markers such as cardiac troponin T (Wu, 1999), and systemic inflammatory markers such as C‐reactive protein (Black et al., 2004).

Prognostic and predictive markers are mainly gene‐based. They describe the natural history of a disease or its response to treatment, respectively, and are mainly used in cancer research. As prominent examples, an over‐expression of the her2/neu gene has been linked to more rapid progression of breast cancer (Olayioye, 2001), and chronic myeloid leukaemia is linked to a genetic abnormality called the ‘Philadelphia chromosome’ (Kurzrock et al., 2003). Next to the search for such individual marker molecules, high‐throughput ‘omics’ technologies could hold a greater promise for the development of ‘intelligent’ diagnostic utilities. In addition, the molecular derivatives of technologies should be supplemented with data regarding enzymatic activities, radiological imaging, as well as behavioural and environmental attributes, in order to reach fully integrated diagnostic utilities (Bostanci, 2020; Nonaka & Wong, 2018). In other terms, at least in the context of systemic health monitoring, we should be leaning towards multi‐dimensional personalized data and biosignatures.

### Systemic biomarkers in dentistry

Biomarker research in dentistry has also been focusing on the investigation of candidate single molecules as diagnostic biomarkers. The best‐known prognostic biomarker is the interleukin‐1 genotype that has been associated with severe periodontal disease (Kornman et al., 1997). Moreover, some research has looked at the influence of endodontic infection on the levels of systemic inflammatory markers (Georgiou et al., 2019). However, also in dental medicine, it is becoming increasingly evident that combinations of host and microbial factors can more accurately serve as diagnostic signatures of the disease, that is biosignatures (Belibasakis & Mylonakis, 2015). In the context of endodontic research on diagnostics (i.e. the topic of this review), on the other hand, the approach may be a different one. There are real methodological issues with systemic biomarkers, as so many confounding factors such as general health may affect the results (Vidal et al., 2016). This is problematic for researchers and clinicians alike trying to develop these areas. Only healthy individuals can be used who are in themselves a subpopulation.

## LOCAL BIOMARKERS AND TARGET FLUIDS

In endodontic research, it may make more sense to search for molecules that are elevated or present locally, that is at or near the site of interest (Table 1). This approach considers the fact that many morphogens are released locally, and merely have endocrine or paracrine function. Moreover, for example in the case of pulpitis in vital pulp treatment, biological information can be gained directly from the affected area or the wound surface. These types of biomarkers, produced locally at the site of interest, have been termed *local biomarkers* (Fitzsimmons et al., 2010). Depending on the clinical question, these biomarkers can be collected non‐invasively or intraoperatively, and again, they can be diagnostic, prognostic or predictive in nature, depending on the clinical problem that is addressed (Table 1).

To collect biomarkers non‐invasively in the oral cavity, saliva is a central favourite medium to collect and study, due to its extreme ease at sampling and plethora of parameters to analyse, including high prediction microbiological and immunological biomarkers for dental caries and periodontal disease (Paqué et al., 2020, 2021), or qualitative detection of antibiotic resistance genes (Belibasakis et al., 2020). Saliva sampling may also conveniently replace the uncomfortable nasal or oropharyngeal sampling for select applications, such as SARS‐CoV‐2 RNA detection, with high specificity and sensitivity (Atieh et al., 2021). However, and despite the great potential of this fluid to monitor systemic conditions and viral infections, saliva is not an easy fluid to deal with (Nonaka & Wong, 2018). Potential analytes may bind to mucins, which can vary rather tremendously between individuals and age groups based on their salivary flow rates (Eliasson & Carlén, 2010). Moreover, saliva is a mixed fluid by its nature, including viscous glycoproteins and prokaryotic and eukaryotic cell ‘contaminants’. Last but not least, saliva contains a mixture of systemic and local biomarkers (Javaid et al., 2016), and may thus be less specific than locally sampled fluids. Nevertheless, one of the best‐known diagnostic biomarkers in periodontal disease, matrix metalloproteinase (MMP)‐8, in a point‐of‐care mouthrinse test showed a good specificity on disease severity and potentially higher sensitivity to clinical surrogate markers (Sorsa et al., 2020).

Fluids to be considered in endodontic biomarker research include dentinal fluid, pulpal fluid (after the arrest of bleeding in vital pulp treatment), pulpal blood, gingival crevicular fluid and periapical fluid (Rechenberg & Zehnder, 2014). In clinical examples, fluids to be assessed in the context of pulpitis treatment include dentinal fluid, pulpal fluid and pulpal blood (Mente et al., 2016; Sharma et al., 2021). In the context of PIDAP, pathology‐specific molecules could be searched for in saliva or gingival crevicular fluid. Alternatively, apical fluid could be collected and screened for in teeth that have already received a (perhaps non‐indicated) root canal cleaning and shaping procedure. In any event, the question as to which fluid could be most suitable to study a certain condition of interest in endodontic research is rarely investigated, and thus represents a wide‐open field for future research. One of the analytical limitations to consider, is that contamination of a collected biological sample with blood from the proximal tissue may interfere with the measurements. This is particularly the case when a protein is measured by ELISA, as the background signals from proteinic blood contaminants can yield erroneously high readouts. Accordingly, when mass spectrometry‐based proteomic analysis is chosen, the high abundance of serum albumin in a sample may ‘mask’ the levels of low‐abundant proteins, which may consequently not be picked up by the analysis (Bostanci & Bao, 2017).

## COLLECTION VEHICLES

Specific collection sites call for different vehicles to collect the target molecules. In periodontal research, the vehicles to collect gingival crevicular fluid have been studied in detail (Guentsch et al., 2011), whilst in endodontic research, there is still some work to do. An important question in that context is to identify collection unit, which shows a good yield, and also releases the molecules under investigation for subsequent analysis (Johnson et al., 1999; Zehnder et al., 2014). Whilst collection papers in the form of absorbent paper strips have been commercially developed for gingival crevicular fluid (example: Periopaper; Oraflow), similar developments are elusive for intracanal collection. Commercially available paper points have been used in many studies. However, their natively high soluble protein content can interfere with proper target molecule quantification, especially in fluids with low yields such as dentinal fluid (Ballal et al., 2017). This limited analyte volume available is a considerable problem in this context, as are the contaminants, threshold/sensitivity of the available assays and the sourcing or true normal/ healthy negative controls (pulpal and apical). A prominent example of these problems is the history of culturing from the root canal via paper points to assess persisting infection, which was abolished after being heavily criticised and found impractical in clinics (Bender et al., 1964). More research is clearly needed to overcome these problems and avoid history repeating itself.

## OUTCOME MEASURES

As indicated above, biomarker studies are usually performed *in vivo*. When searching for possible biomarkers, target proteins could be correlated to the histological appearance of the condition under investigation (Guthrie et al., 1965), which can be an ethical impossibility.

Instead, clear‐cut clinical conditions can be identified, and the biomarker level can then be determined by measurements in the appropriate fluid. The two main scenarios that are conceivable can again be discussed in the context of pulpitis and PIDAP (Table 2):

### Scenario 1

A pre‐treatment condition is not described well enough to know its outcome. An example for that is pulpitis in teeth with deep caries that do not hurt much (Hasler & Mitchell, 1970). Here, marker proteins could be collected from the appropriate fluid to correlate these marker levels to treatment outcome. In this example, the successful treatment outcome is pulp survival after vital pulp treatment (Bjørndal et al., 2019). The predictive value of this marker or these markers can then be scrutinized (Ballal et al., 2022; Sharma et al., 2021).

### Scenario 2

A condition, or its predictability, is not understood from a clinical perspective and biomarker research is pursued to help gain a deeper clinical understanding. In our example, the crevicular fluid of teeth with PIDAP could be compared with counterparts with no pain in the same patient (Yi et al., 2021).

## DATA PRESENTATION AND ANALYSIS

Screening the literature for biomarkers in Endodontology, it becomes apparent that there are multiple issues with data presentation and analysis. Three main points should be emphasized here:

1. Biomarker levels should be *normalized* within the sample. This can either be done by presenting the data against the weight of the collected fluid or by using total protein (TP) as an internal control. Alternatively, when micropipettes are used for collection, fluid volume can be used (Mente et al., 2016). What is frequently done but incorrect is to simply present biomarker weight/ml in the respective assay. Moreover, crude total fluid volume or TP levels should also be presented, so that the reader can assess these values between the test and the control group and the authors can validify the similarity in fluid collection between groups.
2. Biomarker levels between a test and a control condition are frequently compared using statistical hypothesis tests such as t‐test to see if there was a ‘significant difference’ between groups, with the null hypothesis that there is no such difference. However, that kind of analysis is not useful in the context of biomarkers, at least not when their cut‐off value as such, or a value range, is to be targeted. The simplest way to present the data is by a dot plot that shows the overlap in biomarker levels between the test and the control condition or treatment outcome. Subsequently, and if the prognostic value of a biomarker is to be assessed, ROC curves can be constructed based on sensitivity and specificity, and the cut‐off values can be identified by calculating the Youden Index (Youden, 1950). As a reference to these concepts, a recent publication can be consulted, in which pulp survival after direct pulp capping in adult carious teeth was correlated to MMP‐9 levels collected from pulpal fluid (Ballal et al., 2022).
3. In studies recruiting high‐throughput analyses (proteomics, metagenomics etc.), a massive amount of data points become available per sample. The challenge is then how to use this information in a meaningful way for the patient. So far, we have been cataloguing extensively names of proteins and compiling them in lists of core proteomes according to clinical status. Yet, clinical and sample collection design strategies for ‘omics’ studies need to be optimized before their commencement, as there are many technical confounding factors to consider. Guidelines for minimum quality standards in data reporting/deposition need to be drawn and adhered to (Zaura et al., 2021), whereas the respective findings need to be validated in different cohorts and via diverse technical pipelines (Bostanci et al., 2018). High‐throughput data refers not only to proteomics but also to forms of digital (e.g. radiographic) data. Combination of diverse databases via artificial intelligence and machine learning tools are supposed to improve diagnosis, prognosis and treatment planning, whilst minimizing human error (Bostanci, 2020; Hung et al., 2020).

## CHAIRSIDE ASSAYS AND BIOSENSORS

Last but not least, the type of assessment tool that could be developed and used clinically is something that should be addressed. Biosensors have been defined as devices for biomarker assessment in point‐of‐care settings (Mascini & Tombelli, 2008). Ideally, chairside tests to identify local biomarkers in Endodontics should be simple, cheap and straight‐forward. As an example, rapid membrane‐base lateral flow immunoassays could be developed, as has already happened in Periodontology (Heikkinen et al., 2016). These could be used to for example identify individual biomarkers with a predictive value on vital pulp treatment. Because these lateral flow immunoassays merely give a binary result (target molecule present above detection limit yes/no), ideal target molecules are those that are exclusively present in the diseased area. In the example of pulpitis/vital pulp treatment, markers related to neutrophils could be used (Ballal et al., 2022; Rechenberg et al., 2016). Neutrophils are the main cellular drivers of pulp tissue breakdown (Wahlgren et al., 2002), and hardly present under healthy conditions (Nair et al., 2008).

Other simple and potentially useful biomarker assays have been proposed in the form of rapid fluorescence tests in conjunction with paper points (Herzog et al., 2017). Pre‐commercial set‐ups have been used to check whether a root canal system is sufficiently decontaminated to be filled (Knight et al., 2020).

More sophisticated tests for more complex issues could utilize the principles of microfluidics and electrical engineering for analysing metabolites and various other molecules in small volumes of patient‐derived biological samples. Such are Lab‐on‐Chip (Mohammadi et al., 2021) or Lab‐on‐Disk (Mitsakakis et al., 2016) utilities. Routine implementation of such technologies may help us avoid the need for centralised and tedious laboratory‐based analyses, facilitating crucial clinical decision‐making on the spot. Utilities of this kind may deliver useful auxiliary microbiological, immunological or metabolic data that can assist the diagnosis and therefore clinical decision‐making. Examples of molecules that can be detected efficiently by biosensors constitute the nucleic acids, microbial components, glucose, oxygen, carbon dioxide, pH and others, as detailed elsewhere (Mohammadi et al., 2021). In the context of PIDAP, such diagnostic biomarker constellations could be identified in gingival crevicular fluid or saliva to confirm the diagnosis or avoid false treatment.

## CONCLUSIONS

The authors of this text were asked to provide guidelines to improve biomarker research in Endodontics. Facets of diagnostic biomarker research where there is still ample space for improvement were elaborated on via the prism of clinical problems encountered daily in endodontics. Clinical studies should strive at optimizing the sample collection method for the most appropriately chosen analytical method. Searching for the ‘golden biomarker’ may be a simplification, especially in more complex disease states, and biosignature research may be the future. This could ultimately lead to the development of chair‐side diagnostic assays, which benefit clinicians and patients.

## CONFLICT OF INTEREST

The authors have stated explicitly that there are no conflicts of interest in connection with this article.

## AUTHOR CONTRIBUTIONS

M.Z. and G.N.B. wrote the text and approved the final version.
